# High-resolution mapping of centromeric protein association using APEX-chromatin fibers

**DOI:** 10.1186/s13072-018-0237-6

**Published:** 2018-11-16

**Authors:** Eftychia Kyriacou, Patrick Heun

**Affiliations:** 1 Max-Planck Institute of Immunobiology and Epigenetics, Freiburg im Breisgau, Germany; 2 Faculty of Biology, Albert-Ludwigs Universität Freiburg, Freiburg im Breisgau, Germany; 3 Wellcome Centre for Cell Biology, University of Edinburgh, Edinburgh, UK

**Keywords:** Centromere, Chromatin fibers, CENP-A, CCAN, Kinetochore, APEX

## Abstract

**Background:**

The centromere is a specialized chromosomal locus that forms the basis for the assembly of a multi-protein complex, the kinetochore and ensures faithful chromosome segregation during every cell division. The repetitive nature of the underlying centromeric sequence represents a major obstacle for high-resolution mapping of protein binding using methods that rely on annotated genomes. Here, we present a novel microscopy-based approach called “APEX-chromatin fibers” for localizing protein binding over the repetitive centromeric sequences at kilobase resolution.

**Results:**

By fusing centromere factors of interest to ascorbate peroxidase, we were able to label their binding profiles on extended chromatin fibers with biotin marks. We applied APEX-chromatin fibers to at least one member of each CCAN complex, most of which show a localization pattern different from CENP-A but within the CENP-A delineated centromeric domain. Interestingly, we describe here a novel characteristic of CENP-I and CENP-B that display extended localization beyond the CENP-A boundaries.

**Conclusions:**

Our approach was successfully applied for mapping protein association over centromeric chromatin, revealing previously undescribed localization patterns. In this study, we focused on centromeric factors, but we believe that this approach could be useful for mapping protein binding patterns in other repetitive regions.

**Electronic supplementary material:**

The online version of this article (10.1186/s13072-018-0237-6) contains supplementary material, which is available to authorized users.

## Introduction

The centromere is a specialized chromosomal locus that serves as the platform for the assembly of a multi-protein complex known as the kinetochore [1]. The kinetochore constitutes the structural element that mediates the interaction with microtubules, in order to ensure faithful chromosome segregation during every cell division [2]. At its base lies centromeric chromatin containing the centromere-specific histone CENP-A and the constitutive centromere-associated network of proteins (CCAN), and both together form the inner kinetochore. In mitosis, the CCAN serves as a binding platform for outer kinetochore proteins that mediate direct interactions with microtubules [2, 3].

CENP-A is a histone H3-variant and considered to be the epigenetic mark for centromere identity as it has been shown to be sufficient for the specification and the epigenetic propagation of the centromere [4–8]. Centromeric chromatin is composed of interspersed blocks of CENP-A and H3 nucleosomes [9] and is decorated by both euchromatic and heterochromatic chromatin marks, while it is flanked by pericentric heterochromatin [10–12].

The CCAN consists of 16 proteins which can be grouped into five different sub-complexes: the CENP-H/I/K/M, the CENP-L/N, CENP-O/P/Q/U/R, CENP-T/W/S/X complexes, and CENP-C [2, 3]. CENP-C plays a central role in the CCAN since it can directly bind both the CENP-A nucleosome and members of the outer kinetochore complex [7, 13–19]. CENP-N was also shown to directly bind the CENP-A-containing nucleosome, while all the CCAN members together are thought to create a complex meshwork of interactions important both for sustaining the kinetochore structure and function but also involved in the propagation of the epigenetic mark by recruiting factors responsible for the incorporation of new CENP-A [2, 3].

Even though centromeres in most organisms are epigenetically defined, and no specific DNA sequence seems to be sufficient to drive centromere formation, CENP-B is a protein constitutively present at all human and mouse centromeres, except for the Y-chromosome, by binding a specific 17-bp DNA motif, termed the CENP-B box [20–23]. Recent reports highlight the requirement of CENP-B for the stabilization of both CENP-A and CENP-C at centromeres [5, 24, 25]. Notably, CENP-B was also shown to be required for the successful assembly of human artificial chromosomes [26, 27], pointing toward an important role in centromere establishment.

Despite the fact that remarkable advances have been made in understanding the interactions of CCAN members and centromeric chromatin, these have been mainly restricted to the nucleosomal level, namely a view of a dinucleosome H3-CENP-A particle and its interactions with different CCAN members [2]. Other studies have focused on the super-resolution organization of the kinetochore at the highly compacted metaphase chromosomes [28, 29] or extended kinetochore fibers [30]. However, how the CCAN members are organized over the centromeric chromatin domain relative to CENP-A remains unclear. This is mainly due to the repetitive nature of centromeric sequences that does not allow the high-resolution mapping of CCAN and other centromeric proteins at endogenous centromeres using conventional methods, such as chromatin immunoprecipitation (ChIP) followed by DNA sequencing, that rely on annotated genomes.

Here, we developed a novel microscopy-based approach, which we call “APEX-chromatin fibers”, for high-resolution mapping of centromeric proteins onto centromeric chromatin. Using proximity-mediated ligation of biotin, this method allowed us to indirectly map the association sites of these proteins on chromatin fibers in relation to CENP-A. We systematically mapped members of all the CCAN complexes and describe novel localization patterns at centromeres. Importantly, we demonstrate that both CENP-B and CENP-I, unlike all the other tested CCAN members, expand beyond the CENP-A-bound centromere domain. While here we focused on centromeric factors, we believe that our approach could be a useful tool for mapping protein association in other repetitive genomic regions.

## Results

### A novel approach for mapping protein association at repetitive sequences

**Fig. 1:**
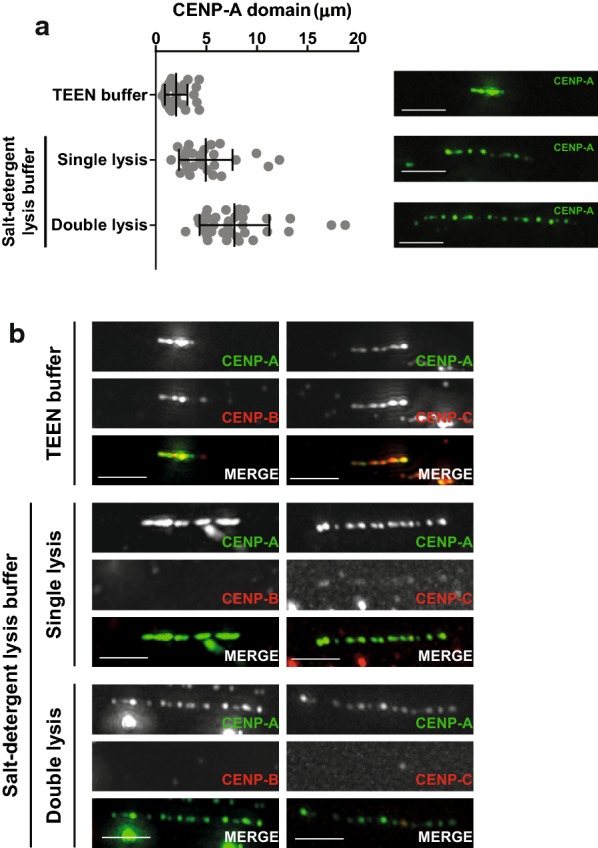
Comparison of different methods for chromatin fiber preparation. **a** Scatter plot depicting the size of CENP-A domain on chromatin fibers prepared with three different methods (left panel). Each dot represents one fiber. Error bars represent mean with SD. *n* = 32, 28 and 37 fibers for TEEN buffer, single lysis step and double lysis , respectively, from one experiment. Representative pictures of fibers (right panel). Scale bar: 2.5 μm. **b** Representative pictures of chromatin fibers prepared with three different methods and stained for CENP-A and either CENP-B or CENP-C. Scale bar: 2.5 μm

The extended chromatin fibers method was proven beneficial for elucidating the organization of centromeric chromatin in interspersed blocks of CENP-A and post-translationally modified H3 nucleosomes [9, 10]. We decided to take advantage of this established method and develop it further in order to decipher the overall spatial organization of centromere-associated proteins over centromeric chromatin. For the preparation of chromatin fibers, a lysis buffer containing high concentration of salt and detergent (salt-detergent lysis buffer) [31] or the less disruptive low-ionic strength TEEN buffer has been used to allow for breaking up nuclei and the subsequent stretching of chromatin fibers [30, 32–34]. In this study, we further introduced an extra lysis step in the salt-detergent buffer prior to fixation and achieved better stretching of the centromeric fiber, since longer stretches of CENP-A were obtained (Fig. 1a). Each of the three methods (TEEN buffer, single and double lysis with salt-detergent lysis buffer) was analyzed for their extent of stretching and preservation of centromere factor staining. While TEEN buffer allows for different centromeric proteins like CENP-B and CENP-C to be retained on the chromatin fiber, it achieves only relatively short fibers, as shown by the domain size occupied by CENP-A (Fig. 1a, b). In turn, the higher degree of stretching and resolution of the double lysis approach comes at the cost of stripping most non-nucleosomal centromere proteins, including the members of the CCAN complexes, from the chromatin fiber, similar to the single lysis using the salt-detergent buffer (Fig. 1b).

**Fig. 2:**
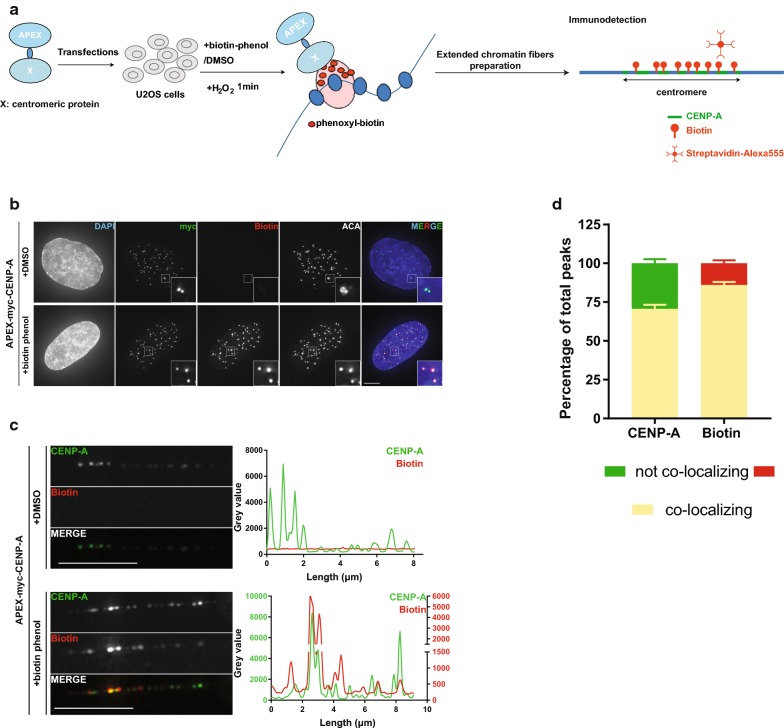
A novel approach for mapping protein binding over centromeric chromatin. **a** Schematic representation of the APEX-chromatin fibers workflow. **b** Representative images of settled U2OS cells expressing APEX-myc-CENP-A, following induction with H_2_O_2_, stained for myc, biotin and centromere marker. Insets represent threefold magnifications of the boxed regions. Scale bar: 5 μm. **c** Representative images of chromatin fibers prepared from cells in **b**, stained for CENP-A and biotin. Scale bar: 5 μm. Intensity plots for CENP-A and biotin gray values along the length of the fiber (in μm) are shown on the right. **d** Mean percentages of total CENP-A or biotin peaks showing co-localization with each other or not. Error bar: SEM. *n* = 42 fibers from four experiments

To overcome this problem and inspired by the DamID method [35], we took advantage of an existing proximity-dependent protein labeling method that employs the enzyme Ascorbate Peroxidase 2 (APEX) and developed a novel microscopy-based approach to map protein–chromatin association, which we call “APEX-chromatin fibers”. More specifically, we fused our proteins of interest to the engineered APEX which oxidizes phenol derivatives, like biotin-phenol, in the presence of H_2_O_2_ [36, 37] and produces highly reactive molecules that can attack electron-rich amino acids of proteins in very close proximity (< 20 nm) [38]. Combined with the extended chromatin fiber preparation, this method should allow to indirectly visualize the localization of proteins of interest fused to APEX by detecting biotinylation of salt-resistant chromatin proteins (Fig. 2a). Therefore, we transiently expressed centromeric proteins fused to myc-APEX (about 28 kDa) in U2OS cells for three days, induced biotinylation through addition of biotin-phenol and H_2_O_2_, and prepared double lysis extended chromatin fibers. Centromeres were marked by staining for CENP-A, and biotinylation was detected with Streptavidin conjugated to Alexa555 (Fig. 2a).

As a proof-of-principle, we started by analyzing APEX-myc-CENP-A and compared its biotinylation pattern to endogenous CENP-A, which itself is retained on chromatin fibers and can be used as a direct reference. We first confirmed that the fusion protein is expressed and correctly targeted to the centromere by IF on interphase cells and mitotic chromosomes, and immunoblotting (Fig. 2b, Additional file 1: Figure S1A,B). Biotinylation was observed specifically at the centromeres only in the presence of biotin-phenol and not DMSO (Fig. 2b, Additional file 1: Figure S1A), indicating that the labeling reaction is spatially restricted to the centromeric region, and that the myc-APEX tag does not influence CENP-A localization. Next, we prepared extended chromatin fibers and compared the CENP-A and biotin profile over the centromere region (Fig. 2c). As expected, no biotinylation was observed in the DMSO-treated preparations, while the biotinylation profile in the biotin-phenol-treated sample revealed a large overlap with the CENP-A staining. For more detailed analysis of the biotinylation profile, we focused on the intensity profile of the staining along the fiber (Additional file 1: Figure S1C, Materials and Methods). We found that about 70% of total CENP-A peaks co-localize with biotin, while 86% of the total biotin peaks co-localize with CENP-A (Fig. 2d). We also analyzed the distribution of the CENP-A-mediated biotin peaks and found that only 12.7% do not co-localize with CENP-A, and a minor fraction (1.3%) are found outside the centromere reference domain (Additional file 1: Figure S1D). Together, the above results show that the biotinylation profile on the chromatin fibers from cells expressing APEX-myc-CENP-A is very similar to the pattern of endogenous CENP-A, despite a noticeable amount (14%) of biotin signals not perfectly co-localizing with CENP-A, which could be considered as the error of the method. Hence, for each APEX fusion protein that we subsequently analyzed, we used the overlap of APEX-CENP-A-mediated biotinylation relative to endogenous CENP-A as the reference for comparison.

In order to address how the CCAN proteins are organized overall over centromeric chromatin, we applied APEX-chromatin fibers and analyzed the resulting biotinylation profiles. For each CCAN complex (CENP-C, CENP-H/I/K/M, CENP-L/N, CENP-O/P/Q/U/R and CENP-T/W/S/X), we mapped at least one member to analyze the number of peaks and how they are distributed over the centromere domain defined by CENP-A, allowing us to group them in different categories of distribution patterns.

### CENP-C, CENP-N and CENP-T APEX fusions are confined inside the CENP-A-bound domain but are differently organized than APEX-CENP-A

**Fig. 3:**
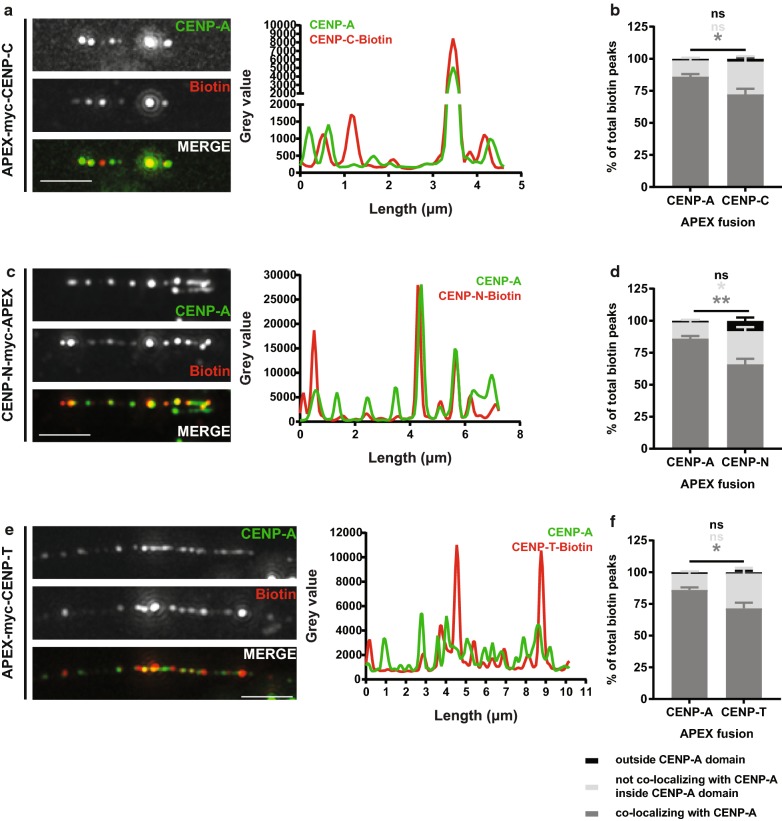
CENP-C-, CENP-N- and CENP-T-APEX are organized differently from APEX-CENP-A but are confined inside the centromere domain. Representative images of chromatin fibers prepared from cells expressing APEX-CENP-C (**a**), CENP-N-APEX (**c**) or APEX-CENP-T (**e**), stained for CENP-A and biotin. Scale bar: 2.5 μm. Intensity plots for CENP-A and biotin gray values along the length of the fiber (in μm) are shown on the right. For presentation purposes, CENP-A gray values in APEX-CENP-T plot were subjected to a fivefold increase. Distribution of biotin peaks compared to APEX-CENP-A (mean with SEM), for CENP-C (**b**), CENP-N (**d**) and CENP-T (**f**). Dark gray: percentage of peaks co-localizing with CENP-A, light gray: percentage of peaks not co-localizing with CENP-A inside the CENP-A-bound domain, black: percentage of peaks found outside the CENP-A domain. FDR adjusted Kolmogorov–Smirnov *p* values are represented as follows: ns for *p* values > 0.05, * for *p* values ≤ 0.05 and ** for *p* values < 0.001. *n* = 42 fibers for CENP-A (from four experiments), 27 for CENP-C (from four experiments), 28 for CENP-N (from three experiments) and 15 for CENP-T (from two experiments)

First, we mapped CENP-C, which lies at the foundation of the kinetochore and is a key component of the CCAN. Following the confirmation that the APEX-CENP-C fusion protein is expressed and correctly targeted to centromeres (Additional file 1: Figure S2A), we analyzed the biotinylation profile on the chromatin fibers in relation to CENP-A (Fig. 3a, b, Additional file 1: Figure S2D). Interestingly, significantly fewer CENP-C-mediated biotin peaks co-localize with CENP-A when compared to APEX-CENP-A (72% vs. 86%, Fig. 2b). This suggests that a fraction of CENP-C localizes at places where CENP-A is absent. In addition, we found that CENP-C is organized in a similar number of peaks as APEX-CENP-A (Additional file 1: Figure S2D), which are confined inside the centromere domain.

We then mapped CENP-N, a member of the CENP-L/N complex, which similarly to CENP-C was also found to be a direct binder of the CENP-A nucleosome [13, 39]. After analyzing the biotinylation profile on chromatin fibers prepared from cells expressing CENP-N-APEX (Fig. 3c, Additional file 1: Figure S2B), we found that like CENP-C, CENP-N is organized in a similar number of peaks as APEX-CENP-A (Additional file 1: Figure S2E). In addition, similar to CENP-C, the CENP-N-mediated biotin peaks do not perfectly co-localize with CENP-A (only 66% of the total peaks shows co-localization with CENP-A) but are also all confined inside the centromere domain (Fig. 3d).

Localizing CENP-T to the centromere has produced different results in different studies. While super-resolution imaging of stretched kinetochore fibers places it closer to the H3 blocks composing centromeric chromatin [30], more recent findings using ChIP followed by sequencing of young α-satellites place CENP-T in the center of the CENP-B box, between two CENP-A nucleosomes [40]. It was also shown that it interacts both with CENP-B and CENP-C [40]. Our analysis of the biotinylation profiles shows that CENP-T is organized in similar number of peaks as APEX-CENP-A (Fig. 3e, Additional file 1: Figure S2C,F) but a smaller portion of those co-localize with CENP-A (72% vs. 86%) (Fig. 3f). Similar to CENP-C and CENP-N, we also find CENP-T to be confined inside the centromere domain but not exclusively where CENP-A is (Fig. 3f).

This defines our first category of association patterns, in which proteins are confined within the CENP-A domain, but reveal a pattern that is distinct from APEX-CENP-A, and includes CENP-C, CENP-N and CENP-T.

### CENP-P, CENP-K and CENP-M APEX fusions are organized similarly to APEX-CENP-A

**Fig. 4:**
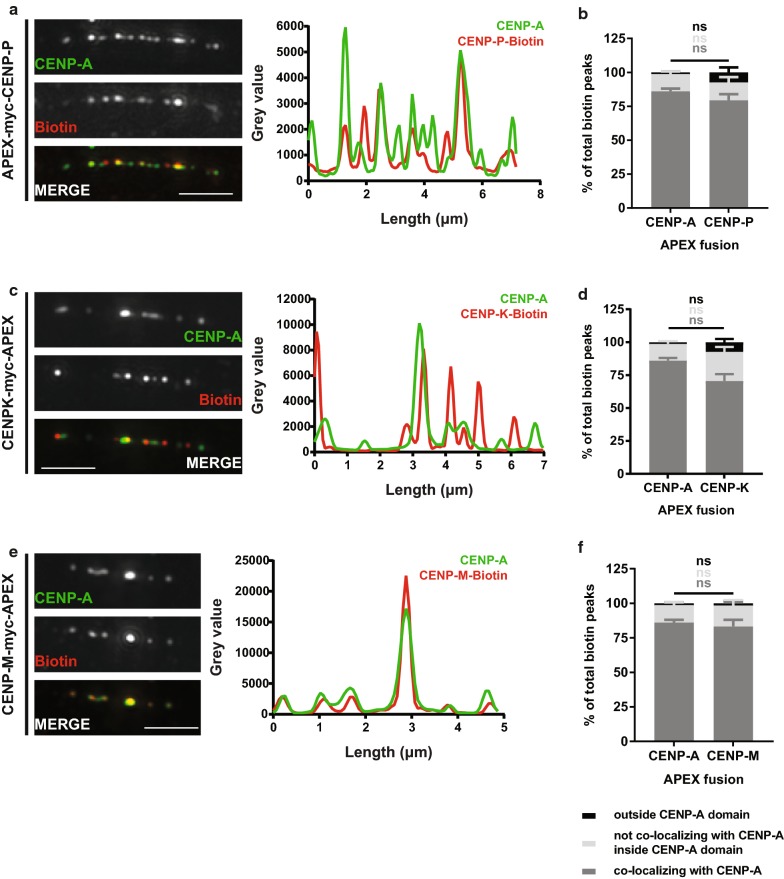
CENP-P-, CENP-K and CENP-M-APEX are organized similarly to APEX-CENP-A. Representative images of chromatin fibers prepared from cells expressing APEX-CENP-P (**a**), CENP-K (**c**) or CENPM-APEX (**e**), stained for CENP-A and biotin. Scale bar: 2.5 μm. Intensity plots for CENP-A and biotin gray values along the length of the fiber (in μm) are shown on the right. For presentation purposes, gray values for CENP-P-biotin were subjected to a twofold increase. Distribution of biotin peaks compared to APEX-CENP-A (mean with SEM), for CENP-P (**b**), CENP-K (**d**) and CENP-M (**f**). Dark gray: percentage of peaks co-localizing with CENP-A, light gray: percentage of peaks not co-localizing with CENP-A inside the CENP-A-bound domain, black: percentage of peaks found outside the CENP-A domain. Not significant FDR adjusted Kolmogorov–Smirnov *p* values > 0.05 are represented by ns. *n* = 42 fibers for CENP-A (from four experiments), 16 for CENP-P (from two experiments), 13 for CENP-K (from three experiments) and 10 for CENP-M (from two experiments)

Other CCAN members presented different distribution profiles than the one discussed above. For the CENP-O/P/Q/U/R complex we focused only on CENP-P. A CENP-P-APEX fusion was transiently expressed in U2OS cells (Additional file 1: Figure S3A) and chromatin fibers were prepared (Fig. 4a). We then analyzed the biotinylation profile of CENP-P and found that it is not significantly different from the biotinylation profile mediated by APEX-CENP-A (Fig. 4a, b, Additional file 1: Figure S3D). In particular, the ratio of biotin to CENP-A peaks is not significantly different between the CENP-P and CENP-A-APEX fusions (Additional file 1: Figure S3D). Similarly, the distribution of the biotin peaks over the centromere domain does not differ significantly (Fig. 4b).

For the CENP-H/I/K/M complex, we mapped CENP-I, CENP-K and CENP-M. CENP-K and CENP-M were also found to be organized in a similar pattern to APEX-CENP-A (Fig. 4c–f, Additional file 1: Figure S3B, C, E, F). In particular, we find that neither the number of biotin peaks mediated by CENP-K and CENP-M nor their distribution is significantly different from the respective number in cells expressing APEX-CENP-A (Additional file 1: Figure S3E-F, Fig. 4d, f).

This defines a second category of distribution patterns, in which proteins are confined within the CENP-A domain and show no significantly different organization from APEX-CENP-A, and includes CENP-P, CENP-M and CENP-K.

### CENP-I and CENP-B extend outside the centromere domain

**Fig. 5:**
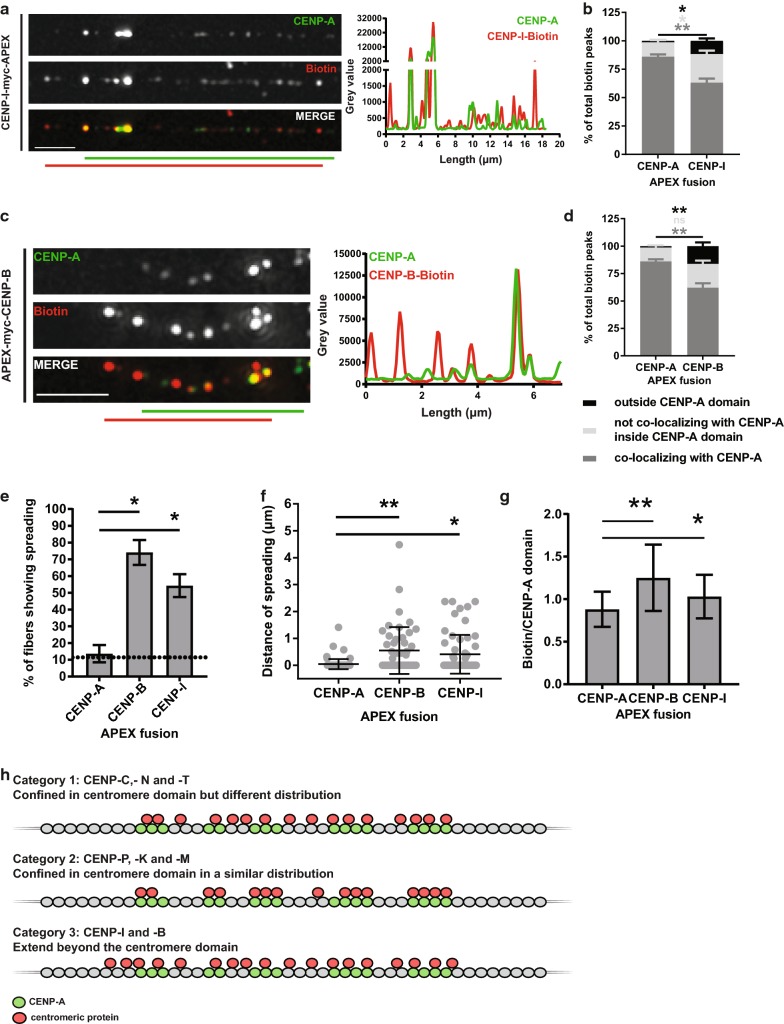
CENP-B- and CENP-I-APEX extend further than the CENP-A-bound centromere domain. Representative images of chromatin fibers prepared from cells expressing CENP-I-APEX (**a**) or APEX-CENP-B (**c**), stained for CENP-A and biotin. Scale bar: 2.5 μm. Intensity plots for CENP-A and biotin gray values along the length of the fiber (in μm) are shown on the right. For presentation purposes, CENP-A gray values in APEX-CENP-B plot were subjected to a fourfold increase. Distribution of biotin peaks compared to APEX-CENP-A (mean with SEM), for CENP-I (**b**) and CENP-B (**d**). Dark gray: percentage of peaks co-localizing with CENP-A, light gray: percentage of peaks not co-localizing with CENP-A inside the CENP-A-bound domain, black: percentage of peaks found outside the CENP-A domain. **e** Mean percentages of total fibers analyzed showing spreading for CENP-A, CENP-B and CENP-I APEX fusion proteins. FDR adjusted unpaired Student’s *t* test *p* values ≤ 0.05 are represented by *. *n* = four experiments for CENP-A and five experiments for CENP-B and CENP-I APEX fusion proteins. Error bars: SD. **f** Scatter plot depicting the distance of spreading of biotin peaks from the CENP-A domain. Each fiber is represented by two dots, for the distance of spreading left and right of the centromere domain. Error bars represent mean with SD. **g** Mean ratio of biotin to CENP-A domain size. Error bars: SD. FDR adjusted Kolmogorov–Smirnov *p* values are represented as follows: ns for *p* values > 0.05, * for *p* values ≤ 0.05 and ** for *p* values < 0.001. *n* = 42 fibers for CENP-A (from four experiments), 24 for CENP-B (from five experiments) and 30 for CENP-I (from five experiments). (H) Summary of the three association pattern categories. The first category includes CENP-C, -N and -T, which are confined inside the centromere domain but are differently organized than APEX-CENP-A. The second category includes CENP-P, -M and -K which are organized similarly to APEX-CENP-A. The last category includes CENP-I and CENP-B, which extend further than the centromere domain

We next focused on the localization of CENP-I on chromatin fibers. After confirming the expression of the APEX-tagged protein and its specific localization at centromeres (Fig. 5a, Additional file 1: Figure S4A-C), we analyzed its biotinylation pattern. Unlike CENP-K and CENP-M of the CENP-H/I/K/M complex, CENP-I exhibited a different distribution profile that falls in neither of the first two categories. First, it is organized in more peaks than APEX-CENP-A over the centromere (Additional file 1: Figure S4D) and a large proportion of biotin peaks does not co-localize with CENP-A inside the centromere (Fig. 5b). Surprisingly, a significant percentage of peaks (11.7%) are also located outside the centromere domain and a large proportion of the fibers analyzed (> 50%) show this “spreading” pattern (Fig. 5b, e).

In addition to the CCAN members, we also explored the high-resolution localization of CENP-B, a DNA-binding protein present at all human and mouse centromeres except for the Y-chromosome. It binds a motif termed the CENP-B box, which is present in the α-satellite sequences [21]. After confirming that the CENP-B-APEX fusion protein is expressed, targeted and is specifically active at centromeres (Additional file 1: Figure S4E,F), we analyzed its biotinylation profile on chromatin fibers (Fig. 5c). Similar to CENP-I, we found that CENP-B is organized in more peaks over centromeric chromatin when compared to APEX-CENP-A (Additional file 1: Figure S4G) and inside the centromere domain not all biotin peaks co-localize with CENP-A (Fig. 5d). Interestingly, on the majority of fibers (> 70%), we find that a significant proportion of CENP-B-APEX-mediated biotin peaks (16.2%) are located outside the centromere domain (Fig. 5d, e).

Our measurements revealed that both CENP-B- and CENP-I-mediated biotin peaks spread significantly further away from the CENP-A domain as compared to APEX-CENP-A (Fig. 5f). In agreement with this, the domain that is occupied by the CENP-I and CENP-B-mediated biotin is significantly larger than the respective domain corresponding to APEX-CENP-A (Fig. 5g).

In summary, by applying APEX-chromatin fibers for mapping protein organization over centromeric chromatin, three different association patterns of centromere factors over centromeric chromatin were revealed (Fig. 5h). In the first category, in which CENP-C, CENP-N and CENP-T fall, the centromeric factors are confined inside the CENP-A-bound centromere domain, but are organized differently than the biotin marks deposited by APEX-CENP-A. In the second category, CENP-P, CENP-M and CENP-K are also confined inside the centromere domain but their pattern is similar to the biotinylation pattern deposited by APEX-CENP-A. In the last category, CENP-I and CENP-B were found to extend beyond the centromere domain.

### Endogenous CENP-B and CENP-I spread further outside the CENP-A-bound domain

**Fig. 6:**
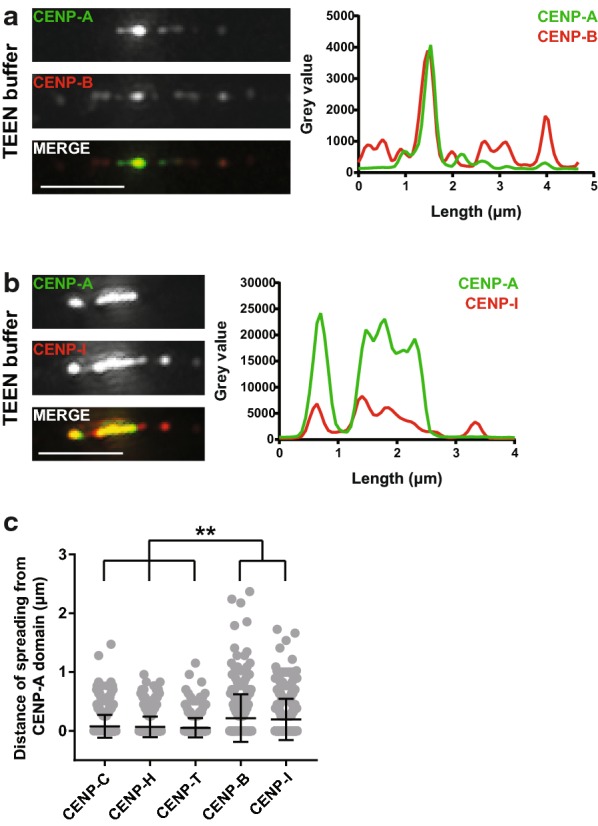
Endogenous CENP-B and CENP-I extend further than the CENP-A domain on TEEN fibers. Representative images of chromatin fibers prepared from U2OS cells using the TEEN buffer and stained for CENP-A and either CENP-B (**a**) or CENP-I (**b**). Scale bar: 2.5 μm. Intensity plots for CENP-A and CENP-B or CENP-I gray values along the length of the fiber (in μm) are shown on the right. **c** Scatter plot depicting the distance of spreading from the edges of the CENP-A domain for CENP-C, CENP-H, CENP-T, CENP-B and CENP-I. Each fiber is represented by two dots, for the distance of spreading left and right of the centromere domain. Error bars represent mean with SD. FDR adjusted Kolmogorov–Smirnov *p* values < 0.001 are represented as **. Not significant *p* values > 0.05 are not depicted. *n* = 142 fibers for CENP-B (from three experiments), 160 fibers for CENP-C (from two experiments), 189 fibers for CENP-H (from two experiments), 177 fibers for CENP-I (from two experiments) and 142 fibers for CENP-T (from two experiments)

To confirm that the “spreading” pattern observed for CENP-B and CENP-I APEX fusions was not a result of overexpression of the transgenic APEX constructs, we prepared fibers using the TEEN buffer that allows the retention of different centromeric proteins (Fig. 1b). By using antibodies against the endogenous proteins, we visualized CENP-B, CENP-I, CENP-C, CENP-H and CENP-T on TEEN fibers (Fig. 6a, b, Additional file 1: Figure S5A-C). In agreement with the “spreading” pattern observed using our APEX approach, we find that the distance that endogenous CENP-B or CENP-I spread outside the CENP-A domain is significantly higher than the equivalent distance for CENP-C, CENP-H and CENP-T (Fig. 6c). To test whether transient expression of the APEX-tagged proteins might exacerbate their spreading outside the CENP-A domain, we prepared fibers using the TEEN buffer from cells expressing either CENP-I or CENP-B APEX fusions (Additional file 1: Figure S5D-G). We did not observe a difference in the comparison for CENP-I, indicating that expression of the transgene does not affect its spreading. For CENP-B, we noticed a slight increase in average domain size for CENP-B, suggesting that additional CENP-B has a small effect on expanding further beyond the CENP-A domain than endogenous CENP-B does.

**Fig. 7:**
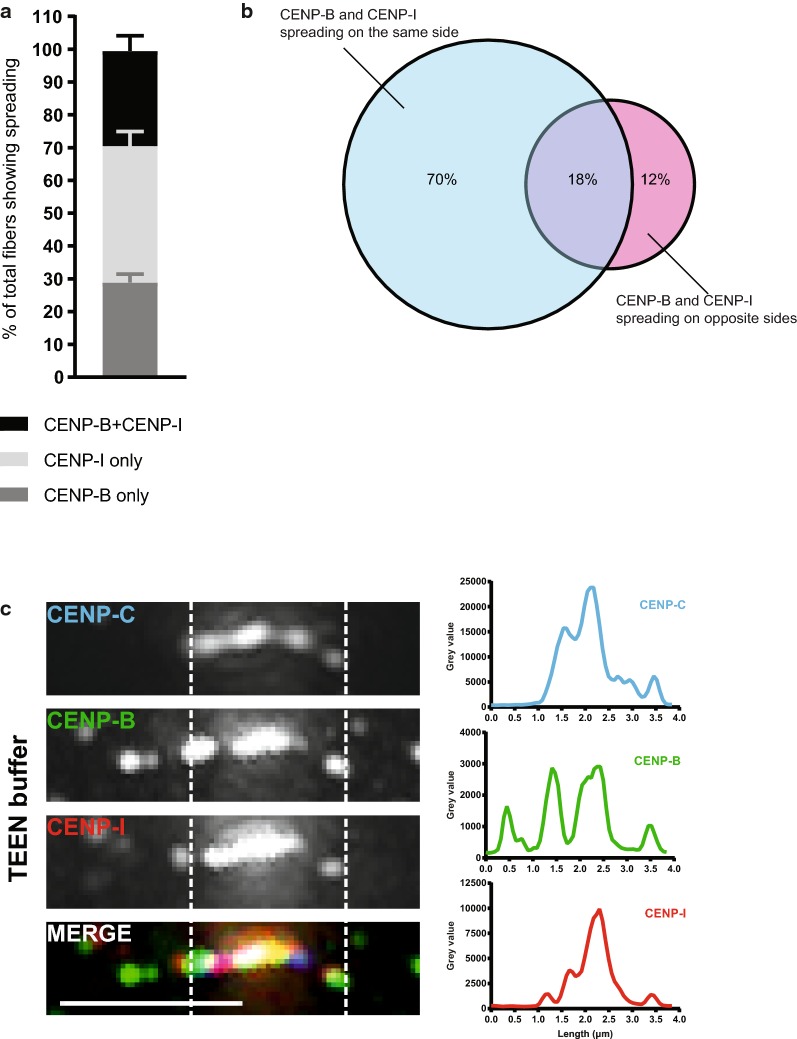
CENP-B and CENP-I are found on the same side when they both spread. **a** Distribution of TEEN fibers showing spreading outside the centromere domain. Mean with SEM. Dark gray: fibers where only CENP-B spreads, light gray: fibers where only CENP-I spreads and black: fibers where both CENP-B and CENP-I spread. **b** Distribution of fibers where both CENP-B and CENP-I spread outside the centromere domain. Percentage is the mean of three experiments. A total of 182 fibers were analyzed. **c** Image of a fiber stained for CENP-C, CENP-B and CENP-I, showing that CENP-B and CENP-I spread on the same side of the centromere domain. Scale bar: 2.5 μm. Intensity plots for each staining along the length of the fiber (in μm) are shown on the right

To investigate whether CENP-B and CENP-I spreading correlates, we used CENP-C as a marker for the centromere domain and simultaneously stained for both CENP-B and CENP-I. When we analyzed fibers that show spreading outside the centromere domain, we found that only 29% show both CENP-B and CENP-I spreading outside the centromere domain (Fig. 7a). In fact, most fibers show either CENP-B or CENP-I spreading alone (29 and 42%, respectively) (Fig. 7a). Interestingly, in the vast majority of fibers with both proteins spreading outside the centromere domain, CENP-B and CENP-I are found together on the same side of the centromere domain (70%) (Fig. 7b, c). Only in few cases CENP-B and CENP-I are located at the opposite sides of the centromere domain (12%), while for some (18%) the two proteins are located together on one side and one of the two (or both) is also found on the opposite side of the centromere domain (Fig. 7b). This suggests that when both CENP-B and CENP-I spread outside the centromere domain, they tend to localize on the same side.

## Discussion

The extended chromatin fibers method has proven to be very useful to study the structural organization of centromeric chromatin [9, 10, 41]. While some protocols favor stretching of the chromatin fiber and achieve better spatial resolution, their harsher lysis conditions come at the expense of many non-nucleosomal proteins being stripped from the chromatin. Accordingly, gentler lysis conditions (e.g., TEEN buffer) allow the retention of chromatin associated factors but result in shorter fibers. This led us to develop a method that achieves well-stretched chromatin fibers, yet maintains a read-out of protein association on the fiber, using biotinylation of chromatin. After successful establishment of the technique, we went on to elucidate the overall spatial organization of members of the CCAN over centromeric chromatin.

Firstly, we were able to confirm the specificity of our approach by directly comparing the binding patterns of CENP-A and APEX-CENP-A-mediated biotin over centromeric chromatin. As a control, we confirmed that in fixed interphase cells and mitotic chromosomes, biotinylation is restricted to the centromere. As expected, we observe strong overlap of the CENP-A and biotin staining in the chromatin fibers, with 86% of the CENP-A-mediated biotin peaks overlapping with CENP-A. A minor population (14%) of the biotin signals did not perfectly overlap with the CENP-A antibody staining, which could be explained by higher sensitivity in the detection of biotin as compared to CENP-A, and could be attributed as the error of the method. We could further confirm that biotinylation was restricted inside the CENP-A-bound centromere domain, suggesting that unspecific biotinylation is not introduced to the adjacent chromatin.

Having confirmed the specificity of our labeling approach, we mapped at least one member of each CCAN complex. We found biotin deposited by CENP-P, -K and -M to be organized similarly to biotin deposited by APEX-CENP-A over centromeric chromatin. CENP-P and the other members of the CENP-O/P/Q/U/R complex were described to be very close to centromeric chromatin in mitotic kinetochores [29], while all three proteins, CENP-P, CENP-K and CENP-M, were reported to be important for proper kinetochore function but with no specialized role at the centromere. It was only recently that the complex, and in particular CENP-Q, was shown to play an important role in microtubule binding cooperatively with members of the outer kinetochore [42].

Surprisingly, despite the fact that CENP-C and CENP-N were reported to directly interact with CENP-A-containing nucleosomes [13, 17, 39, 43, 44], we found that neither of the two overlaps perfectly with CENP-A inside the centromere domain, but instead are also found in places where CENP-A is not present. Our finding might also point to the possibility that CENP-C or CENP-N interacts with proteins other than CENP-A or the CCAN at centromeric chromatin, which are yet to be identified. CENP-C has been described to possess DNA-binding ability [45, 46]. It is, therefore, possible that following its targeting to the centromere, it can associate with DNA in addition to its interaction with CENP-A nucleosomes, allowing it to be stably bound on centromeric chromatin even in places where there is no CENP-A, e.g., after its distribution to the two sister chromatids following DNA replication. Likewise, CENP-N’s association with other CCAN members [44, 47] might confer stability for binding over centromeric chromatin, despite the absence of CENP-A nucleosomes.

While super-resolution study of chicken kinetochore fibers [30], FRET analyses [48] and ChIP data [49] place CENP-T in close proximity to the H3-rich domains of the centromere domain, we find that a high percentage (72%) of CENP-T peaks co-localize with CENP-A. This could be explained by the fact that the CENP-A signals seen on the chromatin fibers contain several CENP-A nucleosomes [9] and CENP-T was found to co-immunoprecipitate with CENP-A nucleosomes when chromatin was not fully digested [49–51]. In addition, it has recently been shown that in ChIP experiments carried out on young α-satellites, CENP-T constitutes a bridge between adjacent CENP-A nucleosomes [40], suggesting that the two proteins are in close proximity, in agreement with our findings.

Interestingly, we found CENP-B and CENP-I to extend beyond the CENP-A-bound centromere domain. It is unclear what the role of these proteins is outside the CENP-A domain at centromeres, but their localization could be implemented at the boundaries of the boustrophedon model [30], without necessarily affecting the function of the kinetochore.

It has previously been proposed that CENP-B might be present outside the CENP-A-bound centromeric domain, based on biochemical data that suggested that half the amount of CENP-B at centromeres is associated with canonical H3 and not CENP-A nucleosomes on the type-I α-satellite array [52]. CENP-B boxes are regularly distributed across the α-satellite arrays and CREST antibodies, which recognize mostly CENP-B, displayed staining of an entire α-satellite array on low resolution chromatin stretches [20, 53, 54]. In contrast, CENP-A was shown to bind only a fraction of the whole α-satellite array [55–57]. Thus, using an alternative approach, we confirm here the previous notion that CENP-B displays extended binding outside the centromere domain. Its localization beneath the kinetochore during mitosis further suggests that CENP-B has a specialized binding pattern over centromeric chromatin [58]. CENP-B’s role is diverse and among others, it is able to promote heterochromatin formation in ectopically integrated alphoid DNA in mouse cells through interaction with Suv39h1 [27]. Its presence beyond the centromere domain might hint toward a role of CENP-B in regulating the boundary between centromeric chromatin and pericentric heterochromatin. However, it was unexpected that CENP-I extends further than the CENP-A domain. Similar to CENP-B [27], CENP-I has additional roles as compared to the other members of the CENP-H/I/K/M complex, especially in promoting CENP-A assembly [59, 60]. Given a recent report that suggests that new CENP-A is loaded preferentially at the boundaries of the centromere domain, to ensure maintenance of the CENP-A-bound domain size [57], it is intriguing to speculate that CENP-B and CENP-I might be involved in the regulation of this process, since they localize to the boundaries of centromeric domain.

While in this study we focused on understanding the organization of CCAN proteins in relation to CENP-A, it would be very interesting for our approach to be combined with FISH in future experiments. Great efforts are being made in order to assemble human centromeric sequences [61–65], and specific centromere probes have been used in the past to study particular centromeres [56, 66]. These studies could be extended with our protein-mapping approach combined with FISH, to elucidate the organization of each CCAN on centromeres of specific chromosomes. Such a novel combination of methods could also allow exploring whether the asymmetric spreading pattern of CENP-B and CENP-I is chromosome specific, especially since it is not observed in all fibers analyzed. It is noteworthy that CENP-A is also asymmetrically distributed on some human chromosomes, especially the centromere of the X chromosome [55, 57]. It would be therefore interesting to address whether the asymmetric distribution of CENP-B and CENP-I correlates with that. Alternatively, it would be interesting to understand that this asymmetric extension of CENP-B and CENP-I outside the CENP-A bound domain reflects the chromosomes that contain more than one adjacent α-satellite arrays containing CENP-B boxes which are not all bound by CENP-A, like on chromosome 17 [67].

Despite the advantages of our approach, we also acknowledge several limitations involved. Although the extended chromatin fibers method is the only available method for higher resolution mapping of protein binding over repetitive regions, it undoubtedly does not achieve the resolution of ChIP coupled to sequencing methods. Here, we focused on proteins that exclusively localize to centromeres, which can be identified and visualized by the specific presence of CENP-A. For proteins that display broad distribution across the genome, the analysis of the binding patterns can be challenging, in part due to the entanglement of several fibers. Moreover, our mapping was carried out in the presence of the endogenous protein, which possibly reduces the coverage of biotinylation. Indeed, we find that for APEX-CENP-A, a small proportion of CENP-A does not co-localize with biotin, suggesting that there are endogenous CENP-A binding sites not labeled with biotin (Fig. 2d). This can be solved by endogenously tagging the genes of interest with APEX. Additionally, as biotinylation through APEX takes place in a radius of 20 nm, the localization of fusion proteins on chromatin can only be detected if they are sufficiently close to proteins which can be retained on the chromatin fiber. A longer flexible linker between the protein of interest and APEX might help circumventing this limitation. Thus, our approach can be tailored for different experimental contexts and combined with other methods such as FISH, and provide a powerful tool for mapping protein distribution in other repetitive genomic regions.

## Conclusions

In summary, we developed a novel approach that allowed us to map the overall association pattern of at least one member of each CCAN complex over centromeric chromatin. We find that the CCAN is organized in three categories of distribution profiles, in which some present a localization similar to CENP-A, in another proteins are confined inside the centromere domain but are differently distributed than CENP-A, and lastly two proteins were found to extend further than the centromere domain. In this study, we focused on centromeric factors, but we believe that our approach could be applicable for mapping protein distribution in other repetitive regions.

## Materials and methods

### Cell culture and transfections

U2OS cells were grown in DMEM supplemented with 10% FBS and 1% Penicillin–Streptomycin at 37 °C in a 5% CO_2_ incubator. Cells were seeded in 6-well plates, a day prior to transfection at a density of 2.5 − 4 × 10^5^ cells per well. Transfections were performed with Lipofectamine 3000 (Life Technologies) according to the manufacturer’s instructions, using 2.5 μg of plasmid DNA and Opti-MEM I reduced serum medium (Life Technologies). Downstream experiments were performed three days post-transfection.

### APEX induction

APEX induction was carried out according to published methods [37]. Briefly, the cell medium was replaced by complete DMEM containing 500 μΜ final biotin-phenol (Iris biotech) or DMSO (control) and incubated for 30 min at 37 °C in a 5% CO_2_ incubator. The APEX enzyme was induced by addition of 1 mM final H_2_O_2_ diluted in 1XDPBS, in the medium, for 1 min at room temperature (RT). Following the induction, the medium was aspirated and cells were washed three times with quencher solution (10 mM sodium azide, 10 mM sodium ascorbate, 5 mM Trolox in 1XDPBS). Next, cells were washed once with 1XDPBS, trypsinized, counted and used for downstream applications.

### Cytological preparations

Cells were allowed to settle on Poly-lysine coated slides in a humidified chamber at 37 °C, 5% CO_2_ for at least 1 h and were fixed with 3.7% Formaldehyde in 0.1% Triton X-100 in 1XPBS (PBST) for 10 min at RT. For preparation of mitotic chromosomes, following trypsinization and before fixation as described above, 1.5 × 10^5^ cells were treated with Colcemid (1 μg/ml) for 30 min at 37 °C, gently shaking. Cells were then pelleted by centrifugation at 1000*g* for 5 min at RT, and the pellet was resuspended in 500 μl of hypotonic buffer (75 mM KCl) and incubated for 10 min at RT, before it was cytospun into a single-chamber cytospin funnel for 10 min at 900 rpm on high acceleration in a Shandon Cytospin 4 onto a poly-lysine coated glass slide. For preparation of chromatin fibers, 5 × 10^4^ cells were pelleted by centrifugation at 1000*g* for 5 min at RT. The cell pellet was resuspended in 500 μl of hypotonic buffer (75 mM KCl) and incubated for 10 min at RT. Then, they were transferred into a single-chamber cytospin funnel and spun for 4 min at 800 rpm on high acceleration in a Shandon Cytospin 4 onto a poly-lysine coated glass slide. For TEEN fibers, the slides were incubated in TEEN buffer (1 mM Triethanolamine-HCl pH 8.0, 1 mM NaCl, 0.5 mM EDTA) for 30 min, they were slowly pulled out the buffer and fixed in 3.7% Formaldehyde in TEEN buffer. Slides were then washed three times in 1XPBS and processed for indirect immunofluorescence. For extended chromatin fibers preparation using salt-detergent lysis buffer, the slides were quickly transferred into a Coplin jar containing freshly prepared salt-detergent lysis buffer (25 mM Tris, pH 9.5, 500 mM NaCl, 500 mM Urea, 1% Triton X-100, 1 mM PMSF), incubated for 20 min at RT and were then slowly pulled out of the buffer. For the double lysis protocol, slides were then washed in PBST for 15 min at RT and incubated again in fresh salt-detergent lysis buffer for 15 min at RT and were slowly pulled out the buffer. Chromatin fibers were fixed 3.7% Formaldehyde in PBST for 10 min at RT.

### Indirect immunofluorescence

Following fixation as described in each relevant application, the slides were washed once in PBST and then blocked in Image-iT^®^ FX signal enhancer in a humidified chamber at RT for at least 30 min. All antibodies were incubated in a 1:1 mix of PBST and 10% normal goat serum (Life Technologies) overnight at 4 °C in a humidified chamber and were used in 1:50 dilution unless otherwise stated: CENP-A (Abcam-ab13939, 1:100), CENP-B (Abcam-ab25734, Santacruz-sc376283), myc (Abcam-ab9106, 1:100), CREST (Europa Bioproducts, 1:200), CENP-I (Abcam-ab118796), CENP-C and CENP-H (Tatsuo Fukagawa), CENP-T (Ben Black). All antibodies were used in 1:100 dilution on mitotic chromosome preparations. Secondary antibodies coupled to Alexa Fluor 488, 555 and 647 (Invitrogen) or Streptavidin conjugated to Alexa 555 were used at 1:100 dilutions (except Streptavidin Alexa 555 on chromatin fibers, which was used in 1:25 dilution). Counterstaining of DNA was performed with DAPI (5 µg/ml), and coverslips were mounted on the slides with 25 μl of SlowFade^®^ Gold antifade reagent.

### Immunoblots

For preparation of protein extracts, cell pellets were washed once in warm 1XDPBS, counted and then resuspended directly in 2X Tris–Glycine SDS sample buffer (Novex, Life Technologies) in a concentration of 2.5 × 10^4^ cells/μl, sonicated and boiled for 5 min at 95 °C. The samples were loaded on 10 or 15% self-casted SDS–polyacrylamide gels, and blotted on Nitrocellulose membranes. Immunoblotting was conducted with the following primary antibodies: anti-CENP-A (Abcam-ab13939) or CENP-I (Abcam-ab118796), both used in a dilution of 1:1000.

### Microscopy

All IF images were taken as 20–50 z-stacks of 0.2 μm increments, using a 100× oil immersion objective on a Deltavision RT Elite Microscope and a CoolSNAP HQ Monochrome camera. All images were deconvolved using the aggressive deconvolution mode on a SoftWorx Explorer Suite (Applied Precision) and are shown as quick projections of maximum intensity.

### Image analysis and quantifications

All images were analyzed on Fiji software [68]. Quick projections of maximum intensity of the deconvolved images were imported in the software and analyzed as follows: quantifications were manually performed on the intensity profile plots of each staining as described in Additional file 1: Figure S1C. For the generation of the intensity profile plots, a segmented line was manually drawn above the identified fiber. The intensity profile was calculated for each staining separately, and graphs were plotted in Excel. Each peak with a gray value ≥ 300 was marked as a peak for all experiments, except experiments with TEEN fibers where the threshold for antibody signals was set to 200 (except Streptavidin, for which the signal threshold was 300, as above). The distance between peaks was measured by calculating the difference between the X-axis positions of each marked peak involved in the quantification. A “threshold for co-localization” was set to 0.192 μm (3 pixels) since the average width of dots on the fibers was measured as ~ 4 to 5 pixels and based on the optical resolution limit. For quantifications of the size of fibers in Fig. 1a, a segmented line was manually drawn above the identified fiber and using the “measure” tool on Fiji, the size was determined. At least 10 fibers were analyzed for each condition, pooled from at least 2 experiments, except the experiment in Fig. 1, which was performed only once.

### Statistical analysis

All statistical analyses and graphs construction were performed in GraphPad Prism version 7.02 for Windows (GraphPad Software, La Jolla California USA, www.graphpad.com). Before performing any analysis, datasets were checked for normality by performing a Shapiro–Wilkinson normality test. Parametric or nonparametric tests were performed accordingly, as indicated in each figure. *P* values were adjusted in *R* using the FDR method and are indicated in each figure. The Venn diagram (Fig. 7b) was generated on http://jura.wi.mit.edu/bioc/tools/venn.php.

## Additional file

**Additional file 1.** **Figure S1.** (A) Representative images of mitotic chromosomes from untransfected U2OS cells or cells expressing APEX-CENP-A following induction with H_2_O_2_, stained for myc, biotin and CENP-A. Insets represent threefold magnifications of the boxed regions. Scale bar: 5 μm. (B) Immunoblot of protein extracts from cells transiently expressing APEX-CENP-A and untransfected U2OS cells, using an antibody against CENP-A. The bottom panel shows Ponceau staining of the blot. (C) Schematic for the analysis of plot profiles of extended chromatin fibers prepared with salt-detergent lysis buffer. Hypothetical intensity plot (endogenous CENP-A in green, biotin in red). The dashed gray line depicts the gray value = 300 threshold. (i) Each peak with a gray value ≥ 300 was accounted. The number of peaks was calculated for each staining. To correct for the size of different centromeres, the ratio of the number of biotin to endogenous CENP-A peaks was always calculated. (ii) Measurement of distances between closest peaks. The green arrow-headed line (dCA) depicts the distance between a CENP-A peak and its closest biotin peak while the red arrow-headed line (db) depicts the distance between a biotin peak and its closest CENP-A peak. The above distances were measured for each peak. If the distance between two peaks was ≤ 0.192 μm, they were marked as “co-localizing” peaks. These data allowed the calculation of the percentage of total biotin or CENP-A peaks co-localizing. (iii) The size of the domain covered by CENP-A (centromere domain) or biotin was determined by measuring the distance between the first and last peak of each staining. To correct for the size of different centromeres, the ratio of biotin to centromere domain size was always calculated. (iv) For calculating the distance of spreading of biotin peaks outside the centromere domain (ds) the distance of the furthest biotin peak from the first CENP-A peak (left and right) was measured. If ds was ≤ 0.192 μm (co-localizing with CENP-A) or if no peaks were found outside the centromere domain, ds was set to zero. (D) Distribution of biotin peaks from cells expressing APEX-CENP-A (mean with SEM) which were used as a reference for downstream analyses. Dark gray: percentage of peaks co-localizing with CENP-A, light gray: percentage of peaks not co-localizing with CENP-A inside the CENP-A-bound domain, black: percentage of peaks found outside the CENP-A domain. n = 42 fibers from 4 experiments. **Figure S2.** (A-C) Representative images of settled U2OS cells expressing APEX-CENP-C, CENP-N-APEX or APEX-CENP-T, respectively following induction with H_2_O_2_, stained for myc, biotin and centromere marker. Insets represent threefold magnifications of the boxed regions. Scale bar: 5 μm. (D-F) Mean ratios of the number of biotin peaks to CENP-A peaks on chromatin fibers from cells expressing CENP-C, CENP-N and CENP-T APEX fusion proteins, as compared to APEX-CENP-A. Not significant FDR adjusted Kolmogorov–Smirnov p values > 0.05 are represented as ns. n = 42 fibers for CENP-A (from 4 experiments), 27 for CENP-C (from 4 experiments), 28 for CENP-N (from 3 experiments) and 15 for CENP-T (from 2 experiments). Error bars: SD. **Figure S3.** (A-C) Representative images of settled U2OS cells expressing APEX-CENP-P, CENPK-APEX or CENP-M-APEX, respectively following induction with H_2_O_2_, stained for myc, biotin and centromere marker. Insets represent threefold magnifications of the boxed regions. Scale bar: 5 μm. (D-F) Mean ratios of the number of biotin peaks to CENP-A peaks on chromatin fibers from cells expressing CENP-P, CENP-K or CENP-M APEX fusion proteins, as compared to APEX-CENP-A. Not significant FDR adjusted Kolmogorov–Smirnov p values > 0.05 are represented by ns. n = 42 fibers for CENP-A (from 4 experiments), 16 for CENP-P (from 2 experiments), 13 for CENP-K (from 3 experiments) and 10 for CENP-M (from 2 experiments). Error bars: SD. **Figure S4.** (A,E) Representative images of settled U2OS cells expressing CENP-I-APEX or APEX-CENP-B, respectively following induction with H_2_O_2_, stained for myc, biotin and centromere marker. Insets represent threefold magnifications of the boxed regions. Scale bar: 5 μm. (B) Representative images of mitotic chromosomes from untransfected U2OS cells or cells expressing CENP-I-APEX following induction with H_2_O_2_, stained for CENP-I, biotin and CENP-A. Insets represent threefold magnifications of the boxed regions. Scale bar: 5 μm. (C) Immunoblot of protein extracts from cells transiently expressing CENP-I-APEX and untransfected U2OS cells, using an antibody against CENP-I. The bottom panel shows Ponceau staining of the blot. (D,G) Mean ratios of the number of biotin peaks to CENP-A peaks on chromatin fibers from cells expressing CENP-I or CENP-B APEX fusion proteins, as compared to APEX-CENP-A. FDR adjusted Kolmogorov–Smirnov p values ≤ 0.05 are represented by *. n = 42 fibers for CENP-A (from 4 experiments), 24 for CENP-B (from 5 experiments) and 30 for CENP-I (from 5 experiments). **Figure S5.** (A-C) Representative images of chromatin fibers prepared from U2OS cells using the TEEN buffer and stained for CENP-A and either CENP-C (A), CENP-H (B) or CENP-T (C). Scale bar: 2.5 μm. Intensity plots for CENP-A and CENP-C, CENP-H or CENP-T gray values along the length of the fiber (in μm) are shown on the right. (D, F) Representative images of chromatin fibers prepared using the TEEN buffer from untransfected U2OS cells and cells expressing CENP-I-APEX (D) or APEX-CENP-B (F) following induction with H_2_O_2_ in the presence of biotin phenol, and stained for CENP-A, biotin and CENP-I or CENP-B, respectively. Scale bar: 2.5 μm. Intensity plots for CENP-A, biotin and CENP-I/B gray values along the length of the fiber (in μm) are shown on the right. (E, G) Scatter plots depicting the size of biotin and CENP-I (E) or CENP-B (G) domain in untransfected U2OS cells on chromatin fibers prepared with TEEN buffer. Each dot represents one fiber. Error bars represent mean with SD. For (E): n = 24 fibers from untransfected U2OS cells (CENP-I) and 38 fibers from cells expressing CENP-I-APEX (biotin) from two experiments. For (G): n = 38 fibers from untransfected U2OS cells (CENP-B) and 40 fibers from cells expressing APEX-CENP-B (biotin) from two experiments. FDR adjusted Kolmogorov–Smirnov p values are displayed in the graphs
