# Supplementary figures and images for: High-resolution mapping of centromeric protein association using APEX-chromatin fibers
Source: Epigenetics Chromatin. 2018 Nov 16;11:68. doi: 10.1186/s13072-018-0237-6 (PMC6238281; doi:10.1186/s13072-018-0237-6)

A

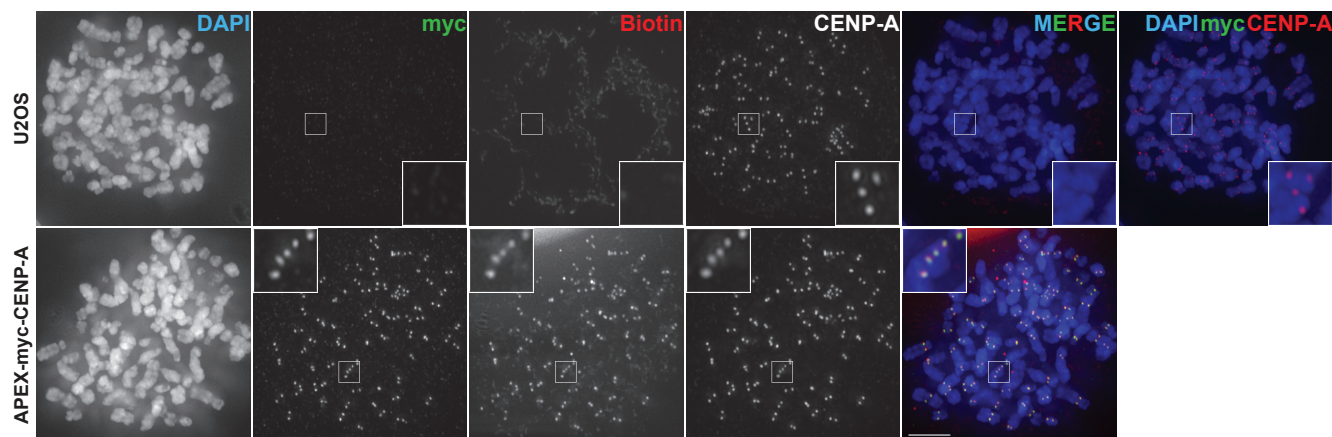

B

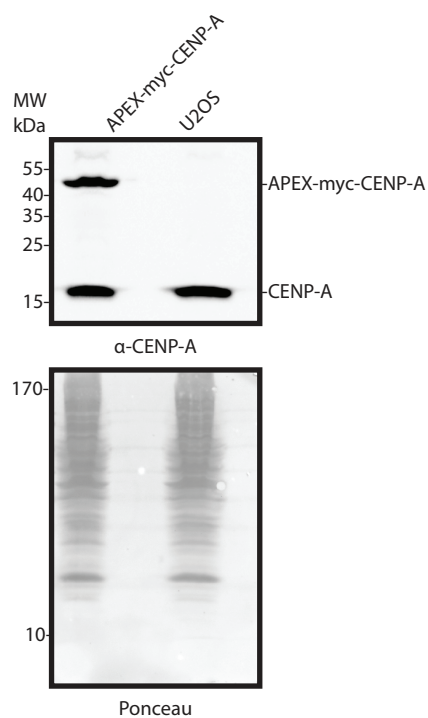

C

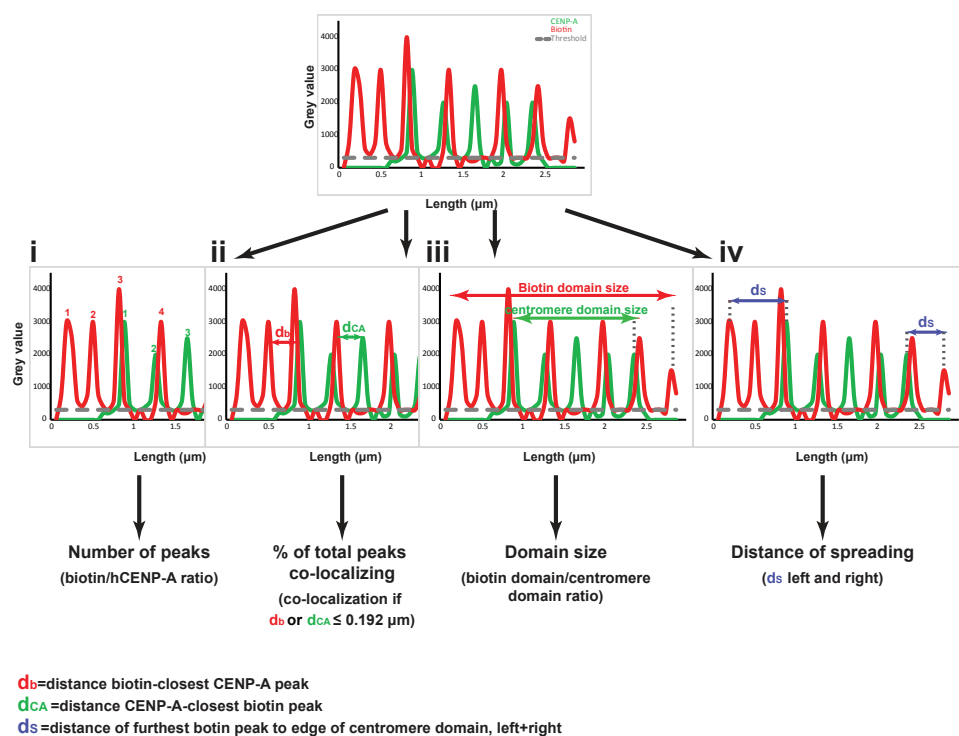

D

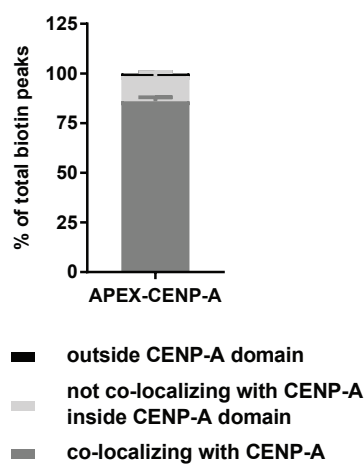

Figure S1

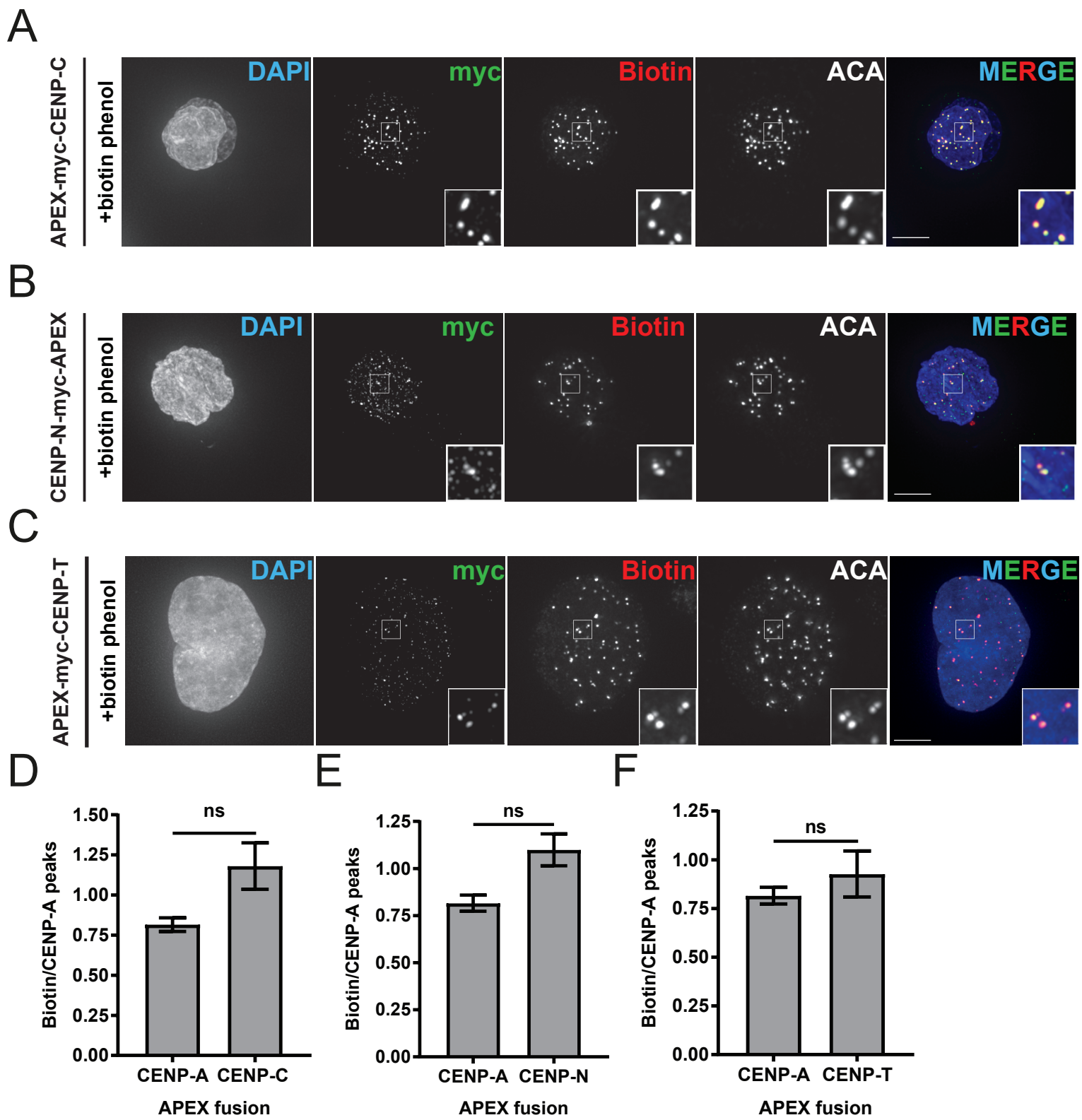

Figure S2

A

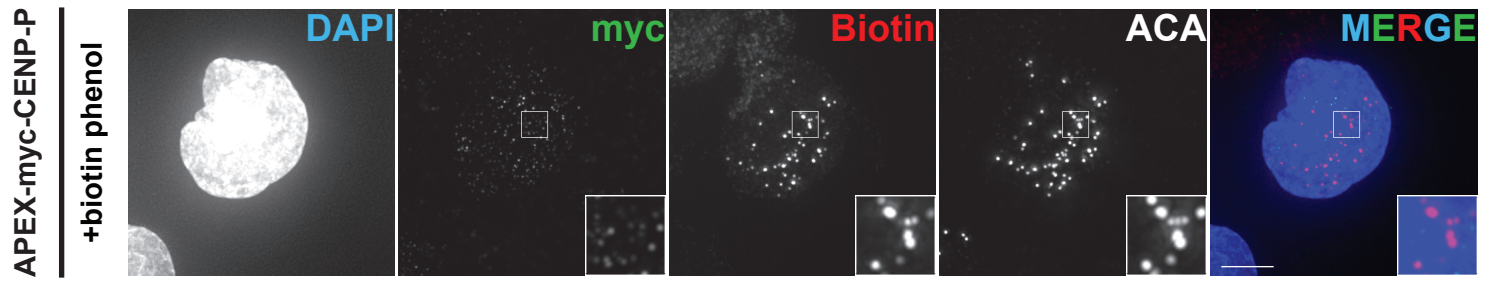

B

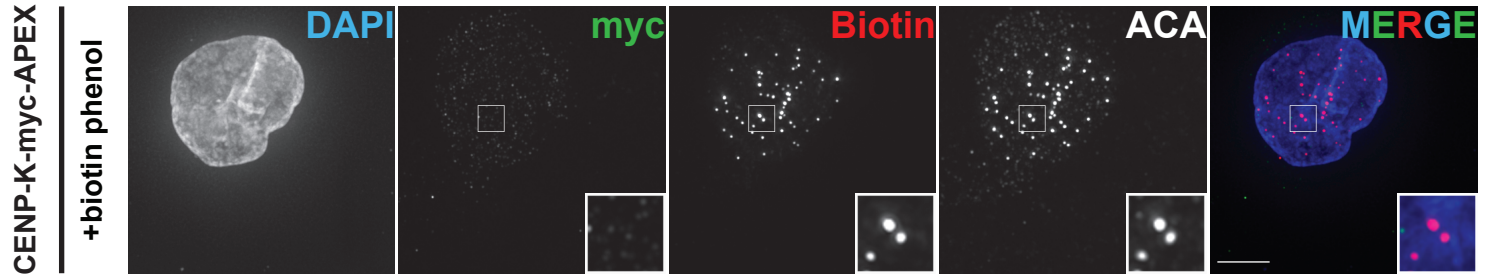

C

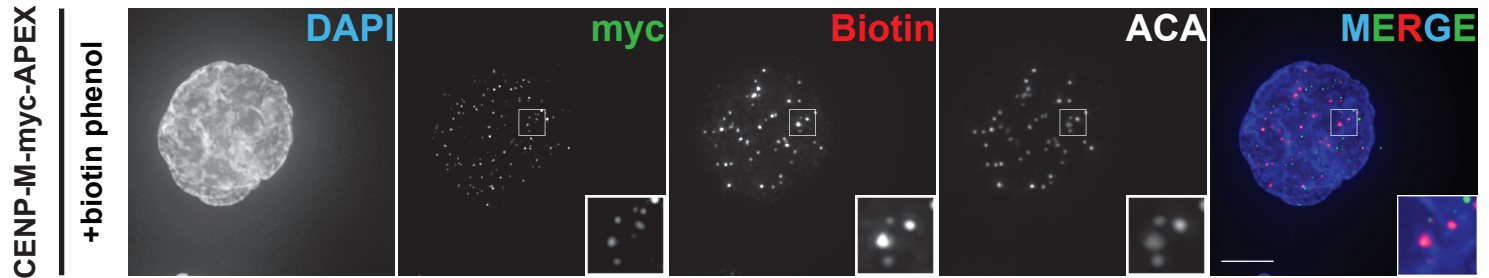

D

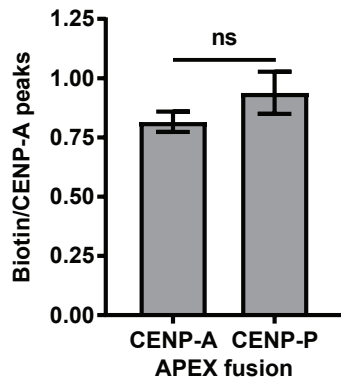

E

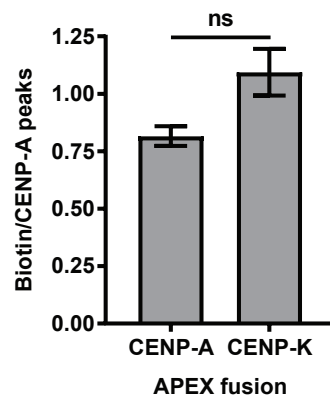

F

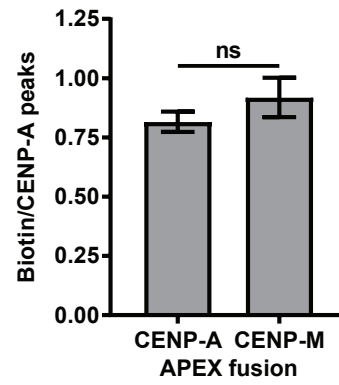

Figure S3

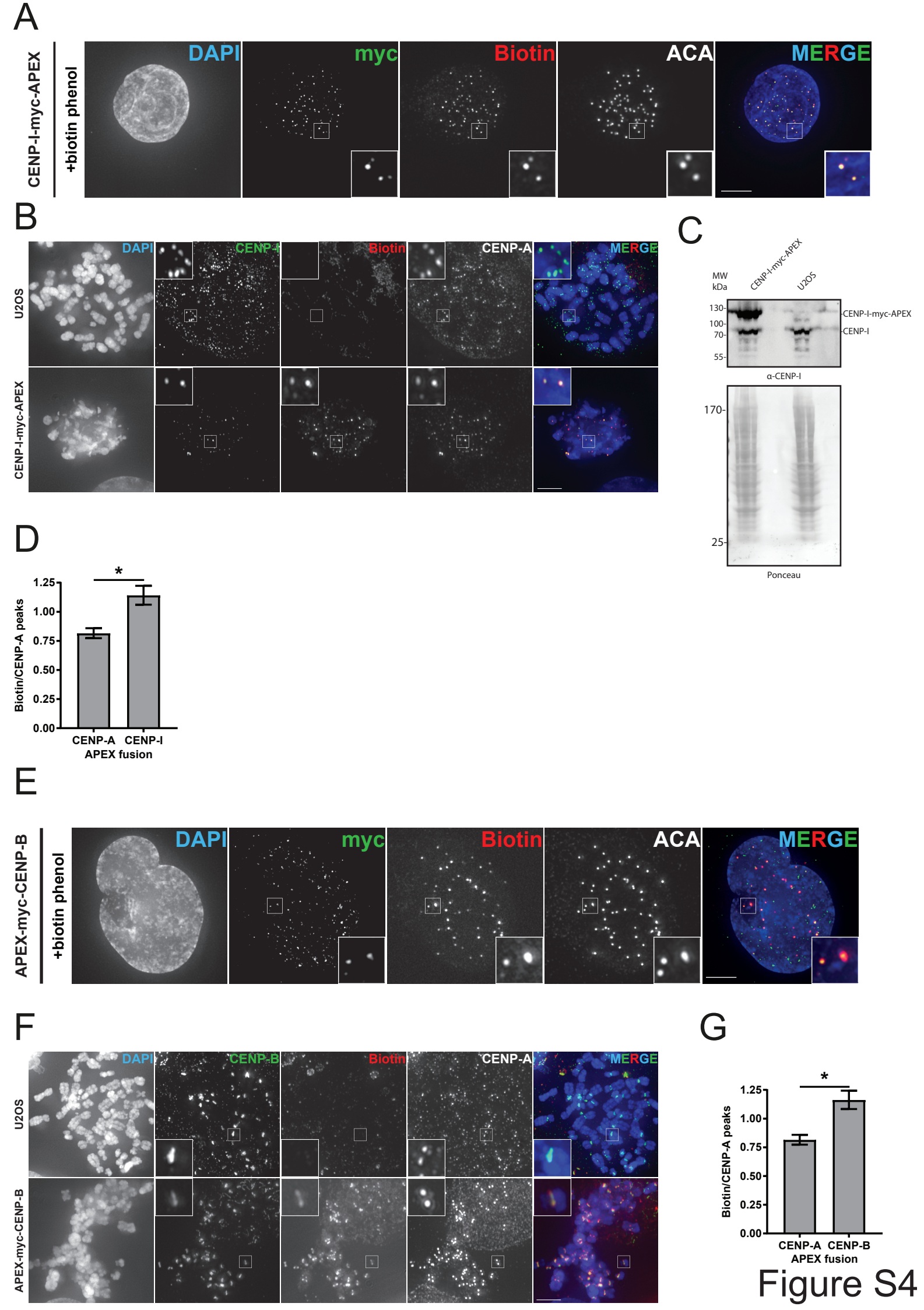

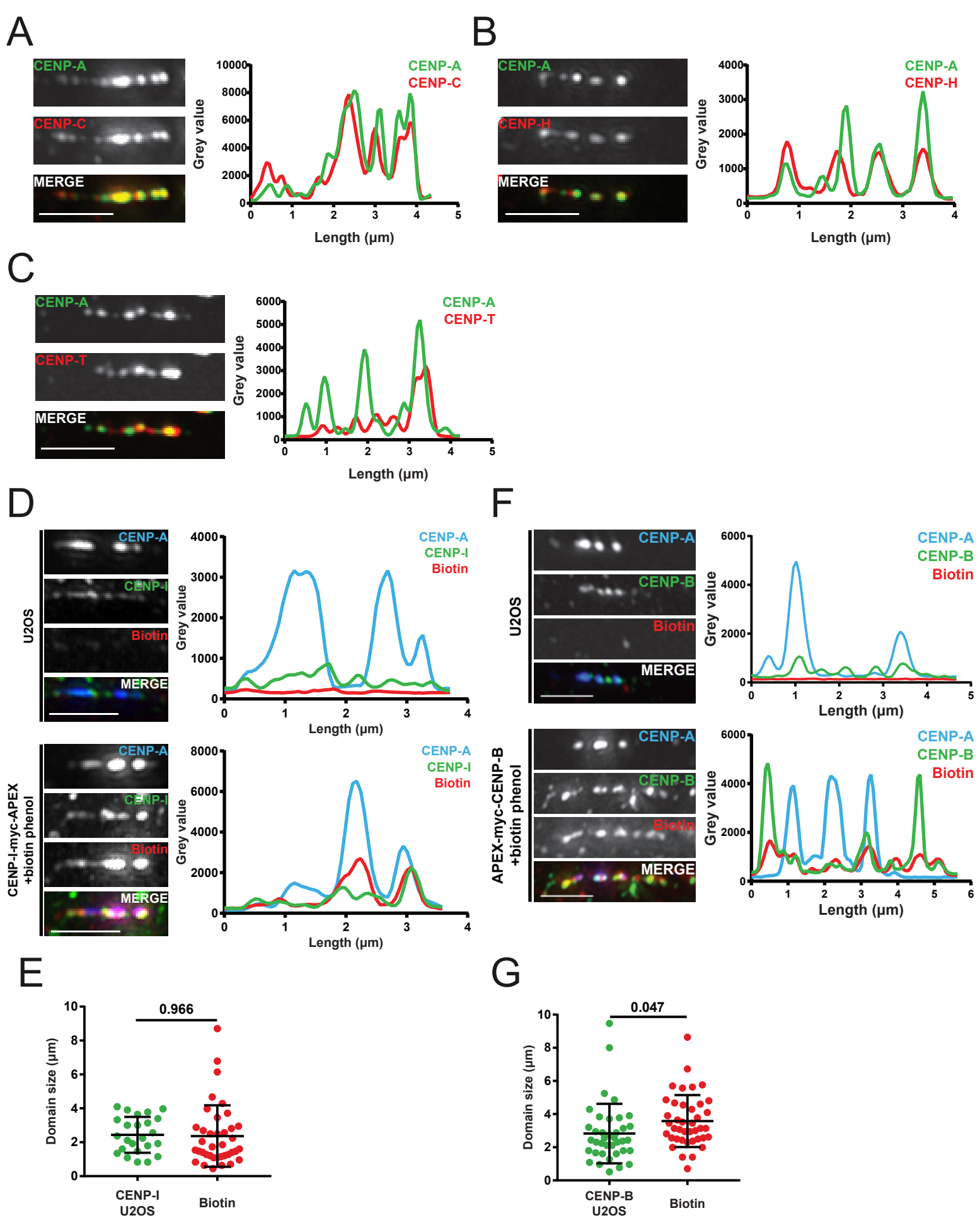

Figure S5

Supplement: Supplementary file 1 — Additional file 1. Figure S1. (A) Representative images of mitotic chromosomes from untransfected U2OS cells or cells expressing APEX-CENP-A following induction with H2O2, stained for myc, biotin and CENP-A. Insets represent threefold magnifications of the boxed regions. Scale bar: 5 μm. (B) Immunoblot of protein extracts from cells transiently expressing APEX-CENP-A and untransfected U2OS cells, using an antibody against CENP-A. The bottom panel shows Ponceau staining of the blot. (C) Schematic for the analysis of plot profiles of extended chromatin fibers prepared with salt-detergent lysis buffer. Hypothetical intensity plot (endogenous CENP-A in green, biotin in red). The dashed gray line depicts the gray value = 300 threshold. (i) Each peak with a gray value ≥ 300 was accounted. The number of peaks was calculated for each staining. To correct for the size of different centromeres, the ratio of the number of biotin to endogenous CENP-A peaks was always calculated. (ii) Measurement of distances between closest peaks. The green arrow-headed line (dCA) depicts the distance between a CENP-A peak and its closest biotin peak while the red arrow-headed line (db) depicts the distance between a biotin peak and its closest CENP-A peak. The above distances were measured for each peak. If the distance between two peaks was ≤ 0.192 μm, they were marked as “co-localizing” peaks. These data allowed the calculation of the percentage of total biotin or CENP-A peaks co-localizing. (iii) The size of the domain covered by CENP-A (centromere domain) or biotin was determined by measuring the distance between the first and last peak of each staining. To correct for the size of different centromeres, the ratio of biotin to centromere domain size was always calculated. (iv) For calculating the distance of spreading of biotin peaks outside the centromere domain (ds) the distance of the furthest biotin peak from the first CENP-A peak (left and right) was measured. If ds was ≤ 0.192 μm (co- [file 13072_2018_237_MOESM1_ESM.pdf]
